# Mechanical and Microstructural Evaluations of Lightweight Aggregate Geopolymer Concrete before and after Exposed to Elevated Temperatures

**DOI:** 10.3390/ma6104450

**Published:** 2013-10-09

**Authors:** Omar A. Abdulkareem, Mohd Mustafa Al Bakri Abdullah, Kamarudin Hussin, Khairul Nizar Ismail, Mohammed Binhussain

**Affiliations:** 1 Center of Excellence Geopolymer & Green Technology (CEGeoTech), School of Material Engineering, Universiti Malaysia Perlis (UniMAP), P.O. Box 77, D/A Pejabat Pos Besar, Kangar, Perlis 01000, Malaysia; E-Mails: mustafa_albakri@unimap.edu.my (M.M.A.B.A.); vc@unimap.edu.my (K.H.); 2 School of Environmental Engineering, Universiti Malaysia Perlis (UniMAP), P.O. Box 77, D/A Pejabat Pos Besar, Kangar, Perlis 01000, Malaysia; E-Mail: nizar@unimap.edu.my; 3 King Abdul Aziz Science & Technology (KACST), P.O. Box 6086, Riyadh 11442, Saudi Arabia; E-Mail: bnhusain@kacst.edu.sa

**Keywords:** elevated temperatures, lightweight geopolymer concrete, thermal shrinkage, compressive strength, geopolymer

## Abstract

This paper presents the mechanical and microstructural characteristics of a lightweight aggregate geopolymer concrete (LWAGC) synthesized by the alkali-activation of a fly ash source (FA) before and after being exposed to elevated temperatures, ranging from 100 to 800 °C. The results show that the LWAGC unexposed to the elevated temperatures possesses a good strength-to-weight ratio compared with other LWAGCs available in the published literature. The unexposed LWAGC also shows an excellent strength development *versus* aging times, up to 365 days. For the exposed LWAGC to the elevated temperatures of 100 to 800 °C, the results illustrate that the concretes gain compressive strength after being exposed to elevated temperatures of 100, 200 and 300 °C. Afterward, the strength of the LWAGC started to deteriorate and decrease after being exposed to elevated temperatures of 400 °C, and up to 800 °C. Based on the mechanical strength results of the exposed LWAGCs to elevated temperatures of 100 °C to 800 °C, the relationship between the exposure temperature and the obtained residual compressive strength is statistically analyzed and achieved. In addition, the microstructure investigation of the unexposed LWAGC shows a good bonding between aggregate and mortar at the interface transition zone (ITZ). However, this bonding is subjected to deterioration as the LWAGC is exposed to elevated temperatures of 400, 600 and 800 °C by increasing the microcrack content and swelling of the unreacted silicates.

## 1. Introduction

Globally, it is well-known that the lightweight concrete (LWC) is more advantageous than the normal weight concrete (NWC) by the reduction of the structure’s dead-weight, better thermal insulation for buildings, and less cost for transportation. Lightweight concretes (LWC) are commonly used in the construction of buildings, bridge deck pavements, and in more limited roles, for entire bridge superstructures [1,2,3]. The usage of lightweight aggregates (LWAs) is one of the most common methods used to produce lightweight building materials. Lightweight aggregate concrete (LWAC) has the obvious advantages of high strength-to-weight ratio, good tensile strength, low coefficient of thermal expansion, and superior thermal and acoustic insulation characteristics due to the air voids within its structure [4].

Recently, geopolymer materials attracted a great deal of consideration as a new environmentally-friendly engineering technology for producing supplementary materials to the ordinary Portland cement (OPC) due to their brilliant mechanical and thermal properties. As compared to the OPC, the geopolymer technology can reduce CO_2_ emission up to 80%–90% [5], and it possesses higher thermal durability in high temperature environments as well [6]. Furthermore, the geopolymer materials have early strength, low permeability, excellent resistance to chemical attacks, good freezing-thawing cycles, and a tendency to immobilize the heavy metal ions in the geopolymeric structure [7,8,9,10,11].

Nevertheless, only few published studies and researches regarding the utilization of LWA in the production of lightweight aggregate geopolymer concrete (LWAGC) has been reported [12]. To our knowledge, there is no other published work yet studying the thermal behavior and thermal durability of these materials at high temperatures. Thus, the first contribution of the current study is to highlight LWAGC preparation using the alkali activation of a locally fly ash (FA) as the only source materials, without any additives. In addition, the main objective of this paper is to experimentally and statistically investigate the mechanical, thermal, and microstructural characteristics of LWAGC materials before and after being exposed to elevated temperatures, ranging from 100 to 800 °C.

## 2. Results and Discussion

### 2.1. Mechanical and Physical Properties of the Unexposed LWAGC

Table 1 illustrates the mechanical and physical properties of the resultant LWAGC at 28 days, excluding the slump value of the fresh concrete, which was measured before the casting process. The LWAGC possesses a compressive strength and unit weight of 18.86 MPa and 1438.7 kg/m^3^, respectively, at 28 days, which permits its classification as a structural LWAC, according to the American Concrete Institute ACI 213R [13]. Furthermore, the resulting LWAGC mechanical strength, with respect to its oven dry (OD)-density is considered as high-quality strength LWAC, when compared with similar structural LWAGCs reported by Yang *et al.* [6]. According to their results, the LWAGC prepared by alkali-activation of ground granulate blast furnace slag (GGBS), by a powder sodium silicate activator and aggregate combination of normal sand and coarse lightweight expanded clay aggregate (LECA), possessed a compressive strength and OD-density of 19.1 MPa of 1615.1 kg/m^3^, respectively, at 28 days. Hence, as they used the same aggregate combinations, the alkali-activation of the FA with a liquid activator appears to be better than the alkali-activated GGBS with a powder activator, as it produces almost the same strength, but with about 11% less density.

**Table 1:** Physical and mechanical strength properties of the resultant lightweight aggregate geopolymer concrete (LWAGC) at 28 days.

Property	LWAGC
Compressive (MPa)	18.86
OD-Density (kg/m^3^)	1438.7
Water absorption (%)	10.7
Fresh Slump (mm)	95

**Figure 1:**
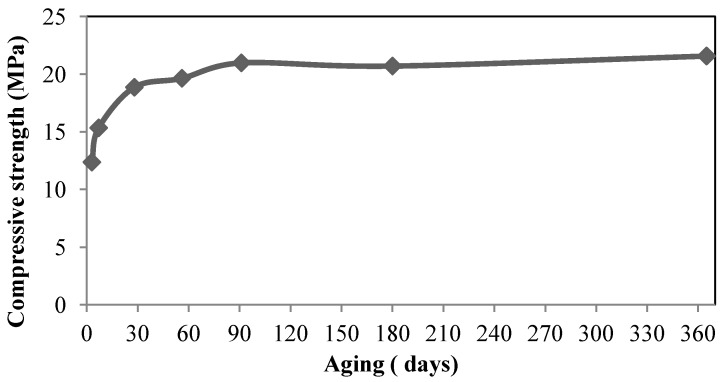
Compressive strength development of the LWAGC *versus* aging times.

Moreover, Figure 1 shows LWAGC strength development *versus* aging times of 3, 7, 28, 56, 91, 180 and 365 days. It can be seen that the LWAGC gains a high rate of strength, increasing at early times of three and seven days, which are equivalent to about 66% and 81% of the 28-day strength, respectively. This trend is attributed to the fact that the geopolymerization process produces excellent early strength at early ages [14]. However, the strength-developing rates are slightly decreased beyond 28 days until the age of 365 days. Collins and Sanjayan [15] reported that the absorbed water and/or activator liquid by the porous aggregates, during the fresh mixing, may have a favorable effect on the strength-development at the long-term ages, due to the continuous activation of the source material (FA), while the moisture and/or absorbed liquid is released from the saturated aggregate. Therefore, the LWAGC is observed to gain compressive strength until the age of 365 days. The results of this experiment show another advantage of the geopolymeric materials, represented by strength-gaining at long-term ages in addition to the advantage of the high early strength reported as early as three and seven days.

### 2.2. Properties of LWAGC Exposed to Elevated Temperatures

#### 2.2.1. Residual Compressive Strength

**Figure 2:**
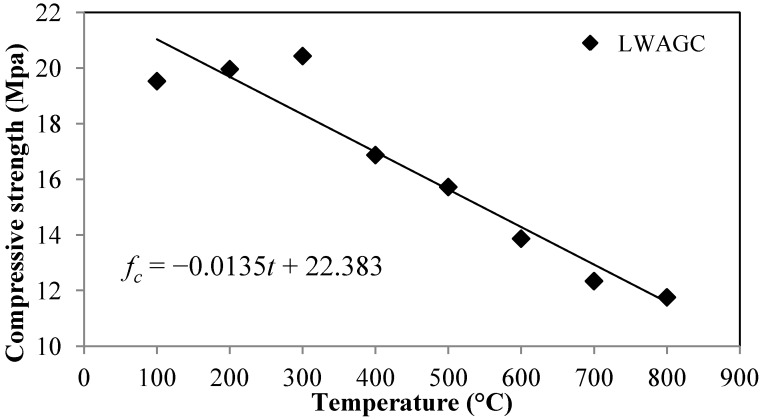
Residual compressive strength of the LWAGCs after being exposing to elevated temperatures of 100 °C to 800 °C.

Figure 2 illustrates the residual compressive strength of the LWAGCs exposed to elevated temperatures ranging from 100–800 °C with incremental steps of 100 °C. It can be seen that the compressive strength of the LWAGC increases after exposure to elevated temperatures of 100–300 °C. The LWAGCs exposed to elevated temperatures of 100, 200 and 300 °C attained relative residual compressive strengths of 103.54%, 105.83% and 108.32%, respectively, as compared to the compressive strength of the unexposed LWAGC (Table 1). The strength-gaining of the exposed FA-based geopolymers to elevated temperatures of 100–300 °C has been already reported in previous investigations [16,17].

Subsequently, the compressive strength of the LWAGC is observed to decrease gradually after being exposed to an elevated temperature of 400 °C, and further up to 800 °C (Figure 2). The strength losses of 13%, 16.6%, 26.7%, 34.6% and 39%, respectively, are reported for the LWAGCs exposed to 400, 500, 600, 700 and 800 °C, respectively.

Furthermore, Figure 2 demonstrates the relationship between the exposure temperatures and the residual compressive strength of the LWAGC, based on the results of 48 LWAGC specimens, exposed to elevated temperatures ranging of 100–800 °C, with incremental steps of 100 °C, which can be fitted as:

(1) $$f_{c}=-0.0135t+22.383$$

where the *f_c_* is the residual compressive strength (MPa) and *t* is the exposure temperature (°C), with a fitting error of (1.1048).

The observed strength loss of the LWAGC exposed to elevated temperatures, higher than 300 °C, and up to 800 °C, is attributed to the essential thermal shrinkage resulted by the loss of mass due to the evaporation of the structural water at elevated temperatures. The water in the geopolymer structure is transformed to a water vapor when the geopolymer is heated above 100 °C, and its pressure increases continuously with the increasing heating temperature [18]. Thus, as the water vapor pressure reaches the maximum limit, the dense geopolymeric matrix, with less permeability characteristics, would be unable of restrain the high thermal stresses, which cause intensive thermal cracks that significantly reduce the compressive strength. This phenomenon is known as the “vapor effect” [18]. In addition, the difference in the thermal expansion between the LWA and the geopolymer paste is another reason behind the strength loss observed for the LWAGC exposed to elevated temperatures of 400–800 °C.

**Figure 3:**
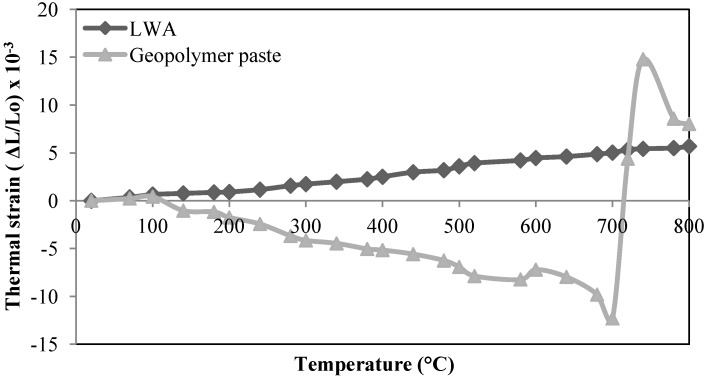
Thermal expansion of lightweight aggregate (LAW) and geopolymer paste.

Figure 3 illustrates the thermal expansion of the geopolymer paste and LWA used in preparation of the LWAGC of this work, at temperatures between 20 and 800 °C, showing the differences in the thermal expansion behavior between the geopolymer paste and the LWA. For the geopolymer paste, a slight expansion is observed as the temperature increases from 70 to 100 °C. Subsequently, the paste undergoes a sharp thermal shrinkage occurred in the temperature range of 100–700 °C, which is related to the evaporation of the structural water [16]. Furthermore, a significant expansion has been detected in the geopolymer paste, instead of the shrinkage experienced earlier, at a temperature range of 700–800 °C. It is likely that a portion of the activating solution remained unreached, or partially reacted, in the geopolymeric matrix during the geopolymer formation [19]. These unreached, or partially reacted, residual species are composed mainly from silicate [20], which experienced extensive thermal expansion at temperature ranges of 700–800 °C, due to the swelling of the high silicate secondary phases as described in references [19,21].

Whereas, the thermal strain data of the LWA presented in Figure 3 shows that the LWA possesses a minimal thermal expansion up to 800 °C. This low thermal expansion of the LWAs is attributed to their low coefficient of thermal expansion (CTE), as they exposed to a pre-heating process at high temperatures during their formation [22]. As a result, the mismatching in thermal expansion of the geopolymer paste and the LWA leads to induce the formation of micorcracks in the interface transition zone (ITZ), as will be discussed in Section 2.2.2. These microcracks, however, decline the compressive strength of the LWAGC. This phenomenon known as ‘thermal inconsistency of the concrete ingredients’ [23,24,25,26].

#### 2.2.2. Microstructure of the Exposed LWAGC

**Figure 4:**
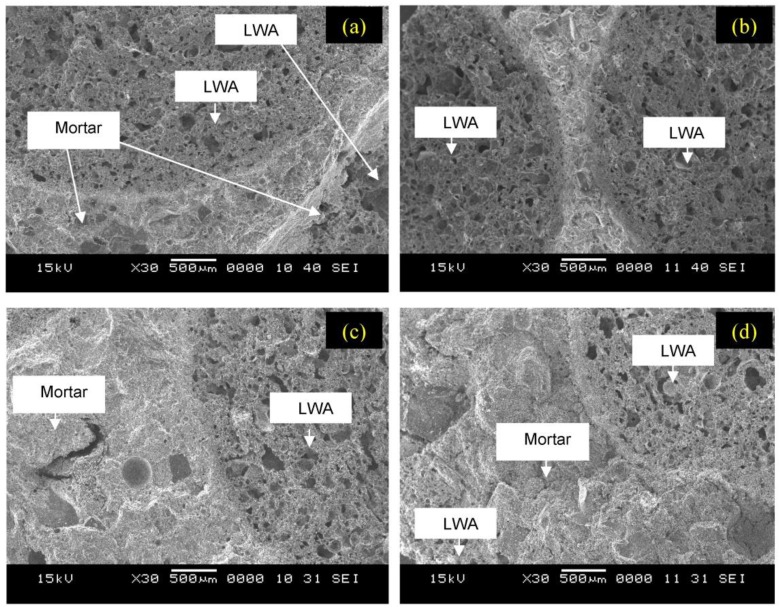
Scanning electron microscope (SEM) micrographs of LWAGCs: (**a**) unexposed; (**b**) exposed to 400 °C; (**c**) exposed to 600 °C and (**d**) exposed to 800 °C.

Figure 4 represents the scanning electron microscope (SEM) micrographs of the unexposed LWAGC, as well as the specimens exposed to the elevated temperatures of 400, 600 and 800 °C. It is observed that the ITZ of the unexposed LWAGC microstructure is indistinct (Figure 4a). This is attributed to the porous nature of the LWAs surfaces, which provides interlocking sites for the geopolymer mortar to form a better interfacial bond between aggregate and mortar at the ITZ [4]. Therefore, it is observed that the geopolymer mortar infiltrates the aggregate surface to a certain depth. This impregnation phenomenon has been also observed by other researchers [25,26].

Moreover, Figure 4b shows the microstructure of the LWAGC exposed to 400 °C. It can be seen that the microstructure appearance does not show many changes from the unexposed concrete (Figure 4a), and appears to be unaffected by this elevated temperature. This can explain the low strength deterioration observed for the LWAGC exposed to 400 °C, shown in Figure 2. On the other hand, the microstructure of the LWAGC exposed to 600 °C, shown in Figure 4c, illustrates the deterioration in the geopolymer mortar due to the high dehydration of the structural water, as well as the formation of microcracks in the ITZ, due to the mismatching in the thermal expansion between the geopolymer paste and LWA. Furthermore, Figure 4d shows the swelling traces of the unreacted silicate phase in the aggregate/mortar bond zone for the LWAGC exposed to 800 °C, as well as the high microcrack content in the aggregate/mortar bond zone.

## 3. Experimental Procedure

### 3.1. Source Materials

The FA used in this work was provided by the Manjung Power Station, Lumut, Perak, Malaysia. The chemical composition of the FA was determined by X-ray fluorescence (XRF) (XRF-Qualitax, Italy) as listed in Table 2. It can be observed that the calcium oxide content is more than 10%; hence it can be classified as Class (C) FA according to ASTM C618-08 [27]. About 90% of FA particles size was smaller than 40 µm, having a specific surface area of 0.463 m^2^/g. The FA was activated with alkaline activator prepared by mixing a technical grade sodium silicate (Na_2_SiO_3_) and sodium hydroxide solution (NaOH). The chemical composition of the Na_2_SiO_3_ was SiO_2_ = 30.1%, Na_2_O = 9.4%, H_2_O = 60.5% and the modulus ratio (*M_S_*) equal to 2 (where *M_S_* = SiO_2_/Na_2_O). The NaOH of 12 M was prepared by mixing sodium hydroxide pellets of 97%–99% purity with distilled water. The alkaline activator prepared by mixing the Na_2_SiO_3_ and 12 M NaOH solution at a constant mass ratio of 1:1.

**Table 2:** Chemical composition of fly ash using X-ray fluorescence (XRF).

Chemical	wt %
SiO_2_	26.4
Al_2_O_3_	9.25
Fe_2_O_3_	30.13
TiO_2_	3.07
CaO	21.6
MnO	0.27
CuO	0.14
K_2_O	2.58
P_2_O_5_	0.67
SO_3_	1.3
SrO	1.57
LOI	3.02

The LWA used for the LWAGC preparation was commercial lightweight expanded clay aggregate (LECA). The LWA granules were spherical in shape with closed surfaces, having a slightly rough and porous texture. The physical properties of the LWA are given in Table 3. The fine aggregate was locally available river sand, having water absorption of 2.2% and specific gravity of 2.50. The sieve analysis for the LWA and sand is shown in Table 4. The aggregate particle distribution for the LWA ranged from 4 to 8 mm, and the maximum size of the sand was 2.38 mm having fineness modulus of 2.83.

**Table 3:** Sieve analysis for the lightweight aggregate (LWA) and river sand.

Sieve (mm)	Passing (%) Sand	Passing (%) LWA
9.5	–	100
6.3	–	60
4.75	100	25
2.38	98.75	0
1.19	95	–
0.59	70	–
0.30	40	–
0.15	10	–

**Table 4:** Physical properties of LWA.

Property	Value
Specific gravity (OD)	0.9
Specific gravity (SSD)	1.05
Apparent Specific gravity	1.07
Density (OD)	897.75 (kg/m^3^)
Density (SSD)	1047.37 (kg/m^3^)
Apparent Density	1067 (kg/m^3^)
Water absorption (%)	17.2

### 3.2. LWAGC Preparation

The mix proportioning of the LWAGC was made according to ACI 211.2 standard [28] and presented in Table 5. The term of water/cement ratio used in the standard was modified to Activator/FA ratio in order to fit the terms used in the geopolymeric technology. In previous work, it has been suggested that the adoption of the proposed Activator/FA ratio of 0.59 by ACI 211.2 standard, produced the optimum preparation and mechanical properties for the LWAGC [12].

**Table 5:** The mix proportion of the geopolymers (kg/m^3^).

Constituents	LWAGC
FA	341.89
Na_2_SiO_3_	100.86
NaOH	100.86
Activator/FA mass ratio	0.59
LWA	484
Sand	823.39
Extra H_2_O	91.47

The LWAs were prepared in oven-dry condition (OD) by being pre-dried at 105 °C for 24 h in order to remove any residual moisture prior to mixing, due to the highly moisture absorption capability of the LWA. Concurrently, the fine aggregate was prepared in air-dry condition (AD) by firstly washing with a tap water and then drying in shade for 24 h prior to mixing. The absorbed water by the LWAs and fine sand was constrained during mixing by adding extra water, equivalent to the quantity of the water absorption of each aggregate type. In practice, the FA and aggregates (LWA + sand) were mixed in a pan mixer for 3 min. Following, the prepared alkaline activator liquid and the extra water dosages were gradually added to the solid constituents. The wet mixing continued for a further 5 min until the mixture homogenized. Subsequently, the slump of the fresh concrete was measured prior to pouring the mixture into 100 mm × 100 mm × 100 mm cubic plastic molds and compacted in three layers using a steel rod to strike each layer 25 times. The molds were sealed from the ambient to prevent the loss of moisture.

### 3.3. Curing Regime

All wrapped molds were cured, undisturbed, in an oven at 70 °C for 24 h after casting. Following, the molds were taken out of the furnace and left to cool at room temperature before demolding. The sealed specimens were stored under ambient conditions.

### 3.4. Elevated Temperatures Exposure Method

Number of LWAGC specimens at 27 days was further exposed to elevated temperatures, ranging from 100 to 800 °C, in increments of 100 °C. The specimens were placed into a furnace and heated at a fixed heating rate of 4.4 °C/min. The geopolymers specimens were kept at each elevated temperatures for 1 h, afterwards, the specimens were left to cool, inside the furnace, to room temperature. Meanwhile, the unexposed specimens were left, undisturbed, at ambient conditions. The compressive strength test for LWAGCs exposed to elevated temperatures of 100 to 800 °C was preformed one day after the heating. Thus, all specimens were left to complete the aging period of 28 days before being tested for their physical and mechanical properties. The density and water absorption of the unexposed geopolymers were preformed according to ASTM C 140-01 [29], and the compressive strength for all specimens was tested according to EN 12390-3 [30]. The density measurements were an average of two specimens, and for the compressive strength, were an average of six specimens.

### 3.5. Dilatometry Analysis

Dilatometry measurements were carried out in order to measure the thermal expansion of the specimens using a Linseis, L75 Laser dilatometer (Sowards, Italy), at temperatures ranging from 20 to 800 °C, with a constant heating rate of 5 °C/min. The test was performed for both geopolymer paste and LWA specimens, as they can present valuable information for the thermal expansion behavior for concrete at high temperatures, as reported in references [16,31]. The test was carried out on 9 mm in diameter ×3 mm in height pellets for specimens sliced from original LWA grains and from 10 mm in diameter ×15 mm in height for geopolymer paste cylinders. The specimens were pre-dried in an oven at 50 °C until they maintained a constant mass [19] before performing the test according to ASTM E 831-03 [32]. The measurements were an average of two specimens. The linear length change values were calculated within the temperature range of 20–800 °C in accordance with the following equations:

(2) $$\varepsilon =\Delta l/l_{0}$$

(3) $$\Delta l=\alpha \cdot l_{0}\cdot \Delta T$$

where ε is the thermal strain (mm/mm), α is the coefficient of thermal expansion (1/°C); Δ*l* is the length change (mm); and *l*_0_ is the initial length of the specimens; Δ*T* is the temperature difference, respectively.

### 3.6. Microstructural Investigation

Scanning electron microscopy (SEM) was performed using SEM JSM-6460 LA Jeol., Tokyo, Japan, in order to investigate the microstructure of the LWAGC, unexposed and exposed, to elevated temperatures. The specimen fragments of the compressive strength tested LWAGCs were mounted in epoxy resin and further vacuumed and coated with a thin layer of platinum using a JFC-1600 auto fine coater Jeol. Japan. The test was carried out using secondary, as well as, backscattered electron (BSE) detectors.

## 4. Conclusion

This paper presents the mechanical and microstructural characteristics of a lightweight aggregate geopolymer concrete (LWAGC) synthesized by the alkali activation of locally sourced FA, before and after exposure to elevated temperatures, ranging from 100 to 800 °C. The mechanical and physical results of the unexposed LWAGC showed a good strength-to-weight ratio when compared with other LWAGCs available in the published literature. In addition, the mechanical strength of the resulting LWAGC showed the possibility of classifying it as a structural LWC that could be used as construction material. Moreover, the continuous gain in the compressive strength of the unexposed LWAGC, with ages of up to 365 days, has added another advantage to these novel materials.

For the exposed LWAGC, the results of the current paper showed that the concrete gained a compressive strength, even after exposure to elevated temperatures of 100, 200 and 300 °C, which made it an excellent refractory material for application when high strength and thermal durability, up to 300 °C, is desired. The strength of the LWAGC started to deteriorate after being exposed to elevated temperatures of 400–800 °C, due to vapor effects, as well as the differences in the thermal expansion between the aggregate and the geopolymeric paste. It was also observed that the rate of strength deterioration was continuously increased with the increase in temperature.

The microstructural investigation by SEM, for the unexposed LWAGC, showed a good bonding between the aggregate and mortar at the ITZ. This bonding, however, was subjected to a continuous deterioration as the LWAGC was exposed to elevated temperatures of 400, 600 and 800 °C, which explains the strength decrease of the LWAGC exposed to these elevated temperatures.
